# Identification and characterization of the causative agents of Focal Ulcerative Dermatitis in commercial laying hens

**DOI:** 10.3389/fvets.2023.1110573

**Published:** 2023-02-08

**Authors:** Diana I. Ayala, Daniel S. Grum, Nicholas P. Evans, Kay N. Russo, Emily A. Kimminau, Benjamin R. Trible, Manohar M. Lahoti, Curtis L. Novak, Theodore P. Karnezos

**Affiliations:** Purina Animal Nutrition Center, Land O' Lakes, Gray Summit, MO, United States

**Keywords:** commercial laying hens, gut microbiota, skin microbiota, FUDS, DFM development

## Abstract

Focal Ulcerative Dermatitis (FUDS) is an emerging dermatological disease that affects cage-free laying flocks, it is characterized by the development of a lesion on the dorsum of the birds; FUDS is sporadic in nature and can result in a drop in egg production and up to 50% of cumulative mortality. A total of two cage-free flocks (flock 1: no history of FUDS; flock 2: birds affected with FUDS) from a commercial laying hen operation in the mid-west U.S. were sampled in this study. The microbial composition of skin, cloacal, cecal, and ileal samples from each bird was characterized through next generation sequencing (NGS). Results identified *Staphylococcus aureus* and *Staphylococcus agnetis* as the potential causative agents of FUDS, being the most predominant in FUDS positive birds. These results were confirmed by plating, with both staphylococci as the only pathogens isolated from lesions of FUDS positive birds. A total of 68 confirmed *Staphylococcus* isolates from skin and environmental samples were further analyzed by whole genome sequencing (WGS) for the presence of antimicrobial resistance (AMR) genes and virulence factors that could have contributed to the development of FUDS. Forty-four-point one-two percent of the isolates had between one and four acquired AMR genes encoding for macrolides, lincosamides, spectrogramines, and beta-lactams resistance. Six classes of virulence factors associated with adherence, enzyme, immune evasion, secretion system, toxin, and iron uptake were identified. The antimicrobial effect of 4 proprietary *Bacillus* Direct Fed Microbial (DFM) combinations was evaluated against the *Staphylococcus aureus* and *Staphylococcus agnetis* isolates, by agar well-diffusion (AWD) assay and competitive exclusion (CE) on broth culture. Through this antimicrobial screening, a particular two-strain combination of *Bacillus pumilus* was identified as the most effective inhibitor of both staphylococci. A customized *Bacillus pumilus* product is being used at different farms with history of FUDS resulting in the successful inhibition of both *Staphylococcus aureus* and *Staphylococcus agnetis*, decreasing FUDS mortalities, and improving harvestable eggs.

## Introduction

In the U.S. consumer and retailer demand on how laying hens are managed has shifted over the past decade, with increased interest in the use of alternative production systems over conventional systems (1). In 2019, 81.6% of egg-laying birds in the U.S. were housed in conventional cages while 13.3% were cage-free (2). On December 1, 2020, the total U.S. cage-free flock had 80.1 million hens, a 9.3 million-head increase from the same date in 2019 (3). These numbers are estimated to increase with over half of the U.S hen housing expected to become cage-free by 2025 (4). Due to the nature of their production design, cage-free housing systems have reportedly higher incidence of bacterial infections (5, 6). Focal Ulcerative Dermatitis Syndrome (FUDS) is one of the emerging diseases cage-free laying flocks are experiencing. FUDS was first described in 2009 in a laying hen operation in Midwest US, the syndrome is characterized by the development of lesions on the dorsum of the birds, just cranial to the uropygial gland. The onset of the condition typically occurs between 24 and 50 weeks of age, lacks seasonality, and is mostly observed in houses with slat floors (7). Outbreaks are relatively sporadic in nature; however, when present can result in up to 50% cumulative mortality.

Typical FUDS lesions are reminiscent of swine exudative epidermitis, a disease caused by *Staphylococcus hyicus*. Staphylococci are ubiquitous in poultry farm environments and are part of the normal skin and mucous membrane microbiota of birds; however, when the integrity of the skin or other mucosal membranes is compromised, some *Staphylococcus* species can become opportunistic pathogens causing localized or systemic infections (8, 9). *S. hyicus* strains have been implicated as secondary causative agents of pre-existing dermatoses (10), they produce exfoliative toxins that induce dermatitis resulting in thickening of the skin (11). Although little research has been done on the etiology of FUDS, a correlation between disease and *S. hyicus* abundance has been observed (7). The production losses due to *Staphylococcus* infections in poultry (egg laying or meat birds) have been associated with lameness, drop in egg production (9, 12), increased mortality, and condemnation of carcasses at slaughter facilities (8).

Antibiotics have been widely used in the poultry industry to promote growth (13) and control pathogens including *Staphylococcus spp.*, thus maximizing production. However, the increased use of antibiotics in livestock production has led to a rapid spread of antimicrobial resistance (AMR) among bacterial isolates and growing public concern about AMR effects in human health (14), resulting in a slow decline in antibiotic use in animal production (13). These trends, together with increased demand by retailers and consumers for antibiotic-free poultry production, has highlighted the importance of developing alternative technologies to reduce/inhibit bacterial pathogens without negative impacts on animal performance.

Direct fed microbials (DFM) have emerged as a viable alternative to the use of antibiotics in poultry farming (15, 16). They are live microorganisms that when fed in adequate amounts confer benefits to the host (17); among the benefits are improved immunity, enhanced growth, and an overall increase in laying performance (14). Multiple bacterial genera have been used in poultry production, with *Lactobacillus, Bacillus*, and *Bifidobacterium* being the most common. Each DFM strain confers a specific action impacting the host and microbial ecology of the gastro-intestinal track. Their mode of action includes the production of antimicrobial compounds (i.e., bacteriocins, organic acids), competitive exclusion mechanisms, and the production of beneficial fermentation products such as volatile fatty acids (18). This study aimed to characterize the cecal, ileal, cloacal, and skin microbiota of FUDS positive and FUDS negative laying hens through next generation sequencing (NGS). The primary goals were to (1) identify the pathogens causing FUDS, (2) isolate the pathogens, (3) identify association among isolates, and 4) develop a DFM combination inhibitory against the FUDS causative agents.

## Methodology

### Flock descriptions and lesion scoring

A commercial laying hen operation in the mid-west United States with a history of FUDS was sampled for this study. Within the operation, two cage-free flocks were selected: (1) flock 1; no history of the disease (Control) and (2) flock 2; birds affected by FUDS. Both flocks originated from the same hatchery, housed in a similar cage-free production system, and were sampled at 73 weeks of age. A total of 20 birds from the control flock and 39 birds from the FUDS affected flock [18 birds showing no symptoms of FUDS (FUDS−) and 21 birds showing visible symptoms of FUDS (FUDS +)] were sampled for this study.

**Figure 1:**
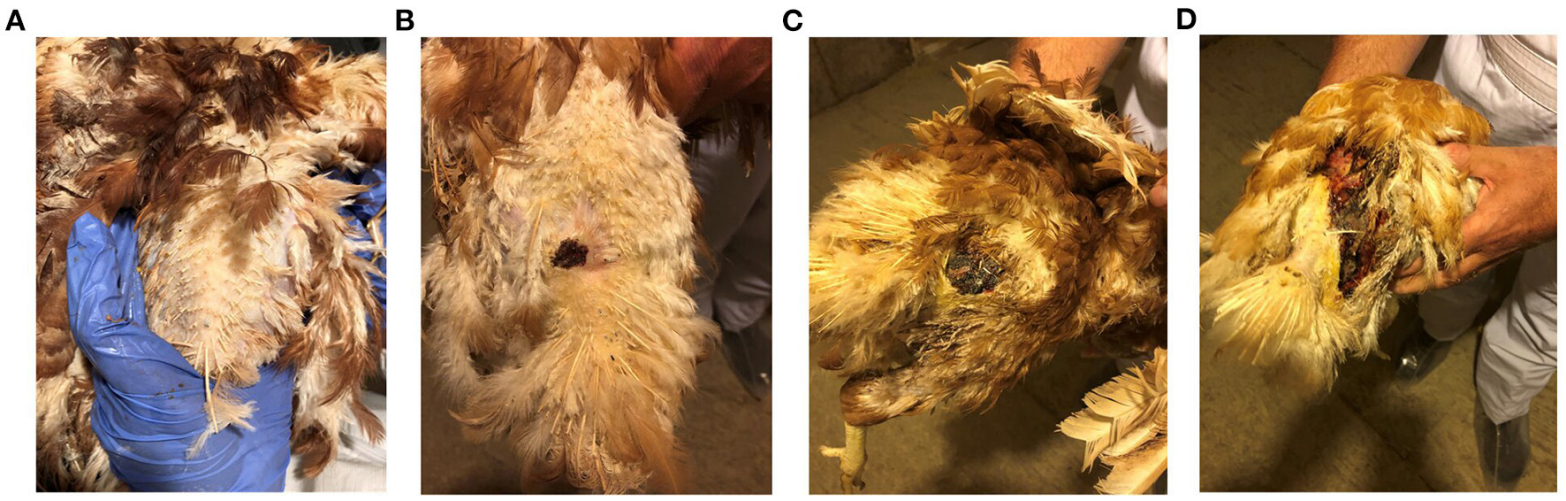
FUDS lesion scoring. Scores were assessed based on size and involvement in the underlying tissue. Scores ranged from 0 to 3: **(A)** 0, no lesion; **(B)** 1, lesion size < 2 cm; **(C)** 2, lesion size 2–6 cm; **(D)** 3, lesions > 6 cm.

Prior to sample collection, birds from the FUDS affected flock were scored for lesions according to the size of the lesion located cranial to the uropygial gland as illustrated in Figure 1. Lesions were assessed for size and involvement of the underlying tissue with scores ranging from 0 to 3: 0, no lesion; 1, lesion size < 2 cm; 2, lesion size 2–6 cm; 3, lesions >6 cm.

### Sample collection for microbiome analysis

A total of four sample types were collected from each bird in this study: skin, cloaca, cecum, and ileum. Samples were collected aseptically to avoid cross contamination. Skin samples were taken using a flocked swab (Puritan, Guilford, ME, USA) that was rotated and swabbed on a 2 cm^2^ area of affected skin cranial to the uropygial gland for FUDS + birds and the same skin area for Control and FUDS–birds. Cloacal samples were collected by inserting a flocked swab ~1 cm into the cloaca, the swab was rotated to allow the collection of enough cloacal material. For cecum and ileum sample collection, the thoraco-abdominal cavity was opened to reveal the gastrointestinal tract. A 1 cm opening was made in the middle of the ileum and a flocked swab was then rotated inside the lumen. The process was repeated with a new swab for the collection of cecal contents. Following sample collection, all swabs were placed into sterile microcentrifuge tubes containing 1 mL of DNA/RNA shield (Zymo Research, Irvine CA), and immediately placed on ice after collection, and shipped overnight to the Emerging Technology Center at Purina Animal Nutrition Center (ETC: PANC) for further processing.

### 16S microbiome DNA extraction and library preparation

Total microbial community DNA was extracted from all sample types and analyzed by using the Quick-DNA Fecal/Soil Microbe miniprep (Zymo Research Corp, Irvine, CA, USA) following manufacturer's recommendations. Pure DNA was quantified using a Qubit 2.0 fluorometer. DNA samples at a 5 ng/μl concentration were used to prepare libraries using the Illumina 16S-metagenomics library preparation protocol. The V3–V4 hyper variable region of bacterial 16S rRNA gene was amplified by using the primers 341F (5′-CCTACGGGNGGCWGCAG-3′) and 805R (5′-GACTACHVGGGTATCTAATCC-3′) containing Illumina adaptors as illustrated in Klindworth et al. (19). The PCR amplicons were checked in agarose gels and purified using AMPure XP beads (Beckman Coulter Inc) as per manufacturer's recommendations. Purified amplicons were then indexed by using the Nextera UD index set (Illumina, San Diego, CA, USA). Libraries were quantified in triplicate using a Qubit 2.0 fluorometer with a dsDNA HS Assay kit (Invitrogen, Carlsbad, CA, USA), pooled at equal concentrations (4 nM) to generate equivalent number of raw reads, and diluted to a final concentration of 6 pM. Amplicon libraries were spiked with 5% PhiX control (Illumina, San Diego, CA, USA) according to manufacturer's recommendations. Samples were sequenced using a MiSeq Reagent kit v3 (600 cycle) on an Illumina MiSeq platform (Illumina, San Diego, CA, USA).

### 16S data analysis and bioinformatics

Raw sequence data were analyzed using quantitative insights into microbial ecology (QIIME2) pipeline (20). Using the MiSeq reporter software (Illumina, San Diego, CA, USA), amplification primers and Illumina adapters were trimmed, samples were demultiplexed, and fastq.gz files generated. Raw sequence data were filtered and processed using the DADA2 pipeline on QIIME2. The 16S rRNA gene sequences were clustered into amplicon sequence variants (ASVs) and taxonomy was assigned based on the comparison against the SILVA database (21). Species richness and diversity indices were calculated using QIIME. Beta diversity was determined using the weighted UniFrac distance with a permutational multivariate analysis of variance (PERMANOVA) to determine differences between microbial communities based on phylogenic relatedness of whole communities.

### Statistical analysis

The linear discriminant analysis (LDA) effect size (LEfSe) (22) was used in this study to identify statistically significant taxa to characterize the differences among FUDS+, FUDS-, and Control groups. The non-parametric factorial Kruskal-Wallis sum rank test was used by the LEfSe method to identify operational taxonomic unit (OTU) abundance differences between two groups. Pairwise tests among groups were then performed by using the Wilcoxon rank-sum test, followed by the LDA on abundances to estimate the effect size of each differentially significant abundant OTU. OTUs were considered significantly different when differences had *p* < 0.05 and an LDA score (log10) > 3.5. A second LEfSe analysis was performed on *Staphylococcus* and *Lactobacillus* to identify specific species associated with the health status of the birds, OTUs were considered significantly different when *p* < 0.05 and an LDA score (log10) was > 3.5.

### Sample collection for bacterial isolation

A total of 10 FUDS+, 10 FUDS-, and 10 Control birds were selected based on the presence/absence of skin lesions for sample collection. Feathers were removed around the preen gland and a 5 cm^2^ surface of the skin was swabbed using pre-moistened sponge swabs (Whirl-Pak, Madison, WI). In addition, a total of 3 environmental sample types, per flock, including from on top of the nest boxes, perches, and scratch areas were collected. A 5 cm^2^ surface was swabbed at an even interval and distributed throughout the houses. Samples were shipped overnight to the ETC: PANC for culture and bacterial isolation.

### Bacterial isolation and growth conditions

Upon arrival at ETC: PANC, samples were homogenized in a stomacher (Stomacher^®^ 400, Seward) for 3 min at 230 rpm. A 1 mL aliquot was collected and added to 9 mL of Baird *Staphylococcus* enrichment broth base (Sigma-Aldrich, St. Louis, MO) supplemented with 0.1 mL Potassium Tellurite solution (Sigma-Aldrich, St. Louis, MO), tubes were incubated aerobically at 37°C for 24 h. After incubation, samples were serially diluted in 9 mL of buffered peptone water (BPW, Sigma-Aldrich, St. Louis, MO), spread plated onto CHROMagar *Staphylococcus* agar plates (CHROMagar, Paris, France), and incubated aerobically at 37°C for 24 h. Based on colony morphology, up to 4 representative colonies were selected from each plate, streaked onto tryptic soy agar plates (TSA, Sigma-Aldrich, St. Louis, MO), and incubated at 37°C for 24 h. A single well-isolated colony was selected from each TSA plate, grown in 9 mL of tryptic soy broth (TSB, Sigma-Aldrich, St. Louis, MO) and incubated at 37°C for 24 h (overnight culture). A 1.5 mL aliquot of the overnight culture was used for gDNA extraction and a 500 μl aliquot was added to 50% glycerol and stored at −80°C for further testing.

### 16S PCR amplification for bacterial speciation

A 1.5 mL aliquot of the overnight culture was used for gDNA extraction using the Invitrogen PureLink DNA extraction kit (Thermo Fisher Scientific, Waltham, MA, USA). DNA samples were quantified using a Qubit 2.0 fluorometer (Life Technologies, CA, United States) and diluted to a 20 ng/μl for PCR amplification.

A 1,483 base pair (bp) region of the 16S ribosomal subunit was PCR amplified using forward primer 5'-AGAGTTTGATCCTGGCTCAG and reverse primer 5'-GGTTACCTTGTTACGACTT. The PCR amplification was carried out using ZymoTaq PreMix according to the manufacturer's directions (Zymo Research, Irvine CA). Following the reaction, PCR products were purified using a DNA Clean and Concentrator kit (Zymo Research, Irvine CA), according to the manufacturer's instructions. Purified products were then sent to the Core Sequencing Facility of the University of Missouri for sanger sequencing. The taxonomic ID for each raw sequence data was assigned based on the results of a National Center for Biotechnology Information (NCBI) basic local alignment search tool (BLAST) inquiry (https://blast.ncbi.nlm.nih.gov/Blast.cgi).

### Whole-genome sequencing

A total of 68 PCR confirmed *Staphylococcus* isolates from the environmental and skin samples of FUDS+ and FUDS- birds were selected for further genotypic analysis. *Staphylococcus* isolates were grown overnight in 9 mL of TSB and incubated at 37°C for 24 h. Total genomic DNA was isolated using the Invitrogen Purelink DNA Extraction kit. Pure gDNA was quantified using a Qubit 2.0 fluorometer and used for library preparation with the Nextera XT v2.0 kit (San Diego, CA, United States) as per manufacturer's recommendations. DNA libraries were paired-end sequenced using the 2 × 300 bp v3 sequencing kit on an Illumina MiSeq platform. Raw reads were preprocessed and filtered using Trimmomatic version 0.36 (23), followed by *de novo* assembly using SPAdes version 11 (24).

### WGS bioinformatic analysis

Assembled genomes were used for the identification of virulence factors and potential antimicrobial resistance (AMR) genes by comparing them to the Virulence Finder and ResFinder databases, respectively (25, 26). Alignments were identified as positive when percentage of identity was 95% or higher. A phylogenetic tree was generated using the concatenated alignment in RAxML (Randomized Axelerated Maximum Likelihood) (27).

### DFMs selection and growth conditions

A total of 4 proprietary *Bacillus* DFM combinations from a set of >500 novel DFMs were selected for this study based on their antagonistic effect against a set of bacterial pathogens including *Salmonella, Staphylococcus aureus, E. coli*, among others. Samples of 1 g of each lyophilized DFM combination were grown individually in 9 mL of Luria-Bertani (LB, ThermoFisher Scientific, Waltham, MA) broth at 37°C for 24 h. Overnight cultures were used for agar-well diffusion assay, as described below, and were serially diluted in 9 mL of BPW and plated aerobically onto LB agar plates (ThermoFisher Scientific, Waltham, MA) at 37°C for 24 h for enumeration.

### Pathogen inhibition screening by agar-well diffusion assay

The agar well-diffusion method, described by Vinderola and others (28) with slight modifications (29), was used to determine the antimicrobial activity of a set of *Bacillus* DFM combinations from a stock culture collection, maintained at ETC: PANC, against *Staphylococcus* isolates. *S. agnetis* and *S. aureus* isolates recovered from the skin of FUDS+ birds were incubated overnight in TSB for 18–24 h at 37°C and then diluted to 10^6^ CFU/mL. A 100 μl of the last dilution was swabbed onto Nutrient agar (Sigma-Aldrich, St. Louis, MO) plates to create a lawn to a final concentration of 10^5^ CFU/mL. Plates were dried for 5 min in a biosafety cabinet, then 6-mm wide wells were made in the agar and each well-duplicate was filled with 100 μl aliquots of one of 4 proprietary *Bacillus* DFM combinations (10^8^ CFU/mL) and a single well with 100 μl of uninoculated TSB broth (control). Plates were incubated at 4°C for 2 h to allow suspensions to diffuse in the agar followed by a 24 h incubation at 37°C. Antimicrobial activity was assessed by measuring the clear zone of inhibition around each well. DFM combinations were ranked for their overall antimicrobial activity [described by Ayala and others (29)] by calculating the sum of the DFM individual inhibition (mm) scores across all *Staphylococcus* isolates tested.

### Competitive exclusion broth culture assay

*Staphylococcus* and *Bacillus* DFM cultures were grown overnight as described above, co-inoculated at 10^5^ and 10^6^ CFU/mL respectively in Nutrient broth (ThermoFisher Scientific, Waltham, MA) and incubated with agitation (130 rpm) at 37°C for 24 h (29). After incubation, co-cultures were serially diluted and plated onto CHROMagar *Staphylococcus* agar plates and incubated at 37°C for 24 h for *Staphylococcus* enumeration. Antimicrobial activity was assessed by the reduction of *Staphylococcus* (log_10_ CFU/mL) with respect to control samples (*Staphylococcus* cultures without DFM) after 24 h of co-inoculation. DFM cultures were ranked for their antagonistic effect by summing the overall reductions (log_10_ CFU/mL) across all *Staphylococcus* isolates tested.

## Results

### Lesion score

Birds from control and FUDS–groups had a lesion score of 0 while birds from the FUDS+ group had lesion scores ranging from 1 to 3 (1 = 14 birds, 2 = 5 birds, and 3 = 2 birds), as shown in Figures 1A–D.

### 16S PCR amplification for bacterial speciation

The taxonomic identification of *Staphylococcus* isolates from environmental samples collected from the Control; and FUDS affected flocks is shown in Table 1. A total of 11 *Staphylococcus* species were recovered from both environments, *Staphylococcus simulans* was the most commonly isolated, found in all environmental samples from both flocks (Table 1).

**Table 1:** Taxonomic identification of *Staphylococcus* isolates from environmental samples collected from the Control and FUDS affected flocks.

**Farm**	**Source**	***S. agnetis***	***S. aureus***	***S. carnosus***	***S. chromogenes***	***S. cohnii***	***S. epidermidis***	***S. haemolyticus***	***S. lentus***	***S. nepalensis***	***S. schweitzeri***	***S. simulans***
Control	Nest box	x	x					x	x			x
FUDS	Nest box	x						x	x			x
Control	Perch		x	x		x			x			x
FUDS	Perch					x	X					x
Control	Scratch	x	x		X						x	x
FUDS	Scratch			x		x				x		x

The taxonomic identification of *Staphylococcus* isolates from skin swabs collected from the Control and FUDS affected flocks is shown in Table 2. A total of 10 *Staphylococcus* species were identified, with skin swabs from non-affected birds showing more *Staphylococcus* species variation. *Staphylococcus aureus* and *Staphylococcus agnetis* were the only two *Staphylococcus* species identified in the FUDS + skin swabs (Table 2).

**Table 2:** Taxonomic identification of *Staphylococcus* isolates from skin swabs collected from the FUDS–and FUDS + birds from control and FUDS affected flocks.

**Farm**	**FUDS status**	***S. agnetis***	***S. aureus***	***S. cohnii***	***S. fleurettii***	***S. lentus***	***S. lugdunensis***	***S. nepalensis***	***S. pasteuri***	***S. sciuri***	***S. simulans***
Control	Negative	x		x		x	x		x	x	
FUDS	Negative	x	x	x	x	x		x		x	x
FUDS	Positive	x	x								

### 16S metagenomics analysis

The complete 16S rRNA data set analyzed in this study is available in the NCBI Sequence Read Archive repository with accession BioProject ID PRJNA884648.

#### Microbial composition by sample type

**Figure 2:**
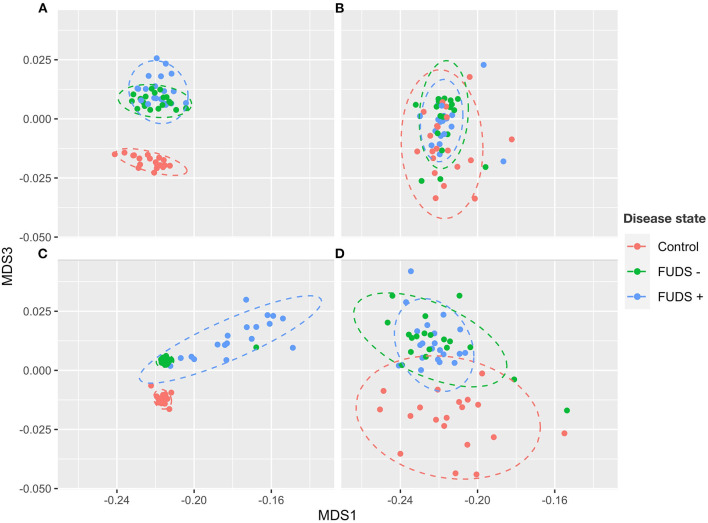
Principal component analysis (PCoA) for Control, FUDS –, and FUDS + flocks by sample type. **(A)** Cecum; **(B)** ileum; **(C)** skin; and **(D)** cloaca. The plot was generated using the Bray-Curtis metrics.

The composition of cecum, ileum, cloaca, and skin bacterial communities were explored through principal coordinate analysis (PCoA) plots using Bray-Curtis index. As shown in the PCoA plot (Figure 2), samples tended to cluster by sample type and sub-clustered by flock of origin with a uniformly divergent microbial composition observed in control and FUDS affected. Within FUDS affected flock, samples clustered by disease state (Figure 2). For the FUDS + birds, the skin samples showed a large deviation from the control and of FUDS–birds, however the ileum samples were not strongly clustered by flock and/or disease state. The weighted UniFrac distance metrics were used to determine the variation among microbial communities from cecum, ileum, cloaca, and skin. In accordance with the PCoA plots, the microbiota from each sample type was found compositionally distinct from the microbiota in other sample types (*p* < 0.001).

#### Relative abundance by phyla

**Figure 3:**
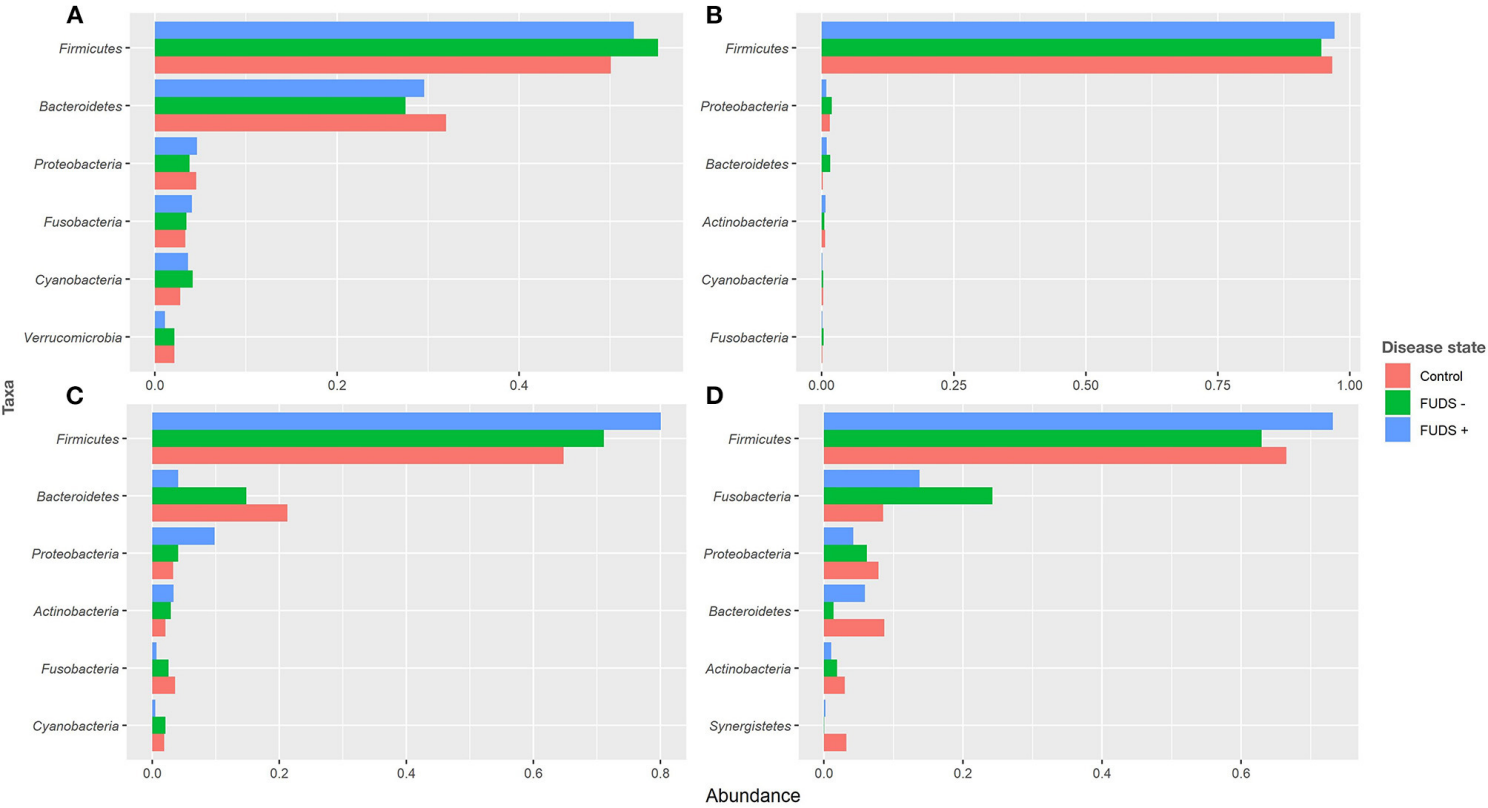
Relative abundance plot of top six taxa at phylum level for Control, FUDS –, and FUDS + flocks by sample type. **(A)** Cecum; **(B)** ileum; **(C)** skin; and **(D)** cloaca.

At the phylum level for all flocks, the six most predominant phyla by sample type are shown in Figure 3. Ileum samples were dominated by *Firmicutes, Proteobacteria, Bacteroidetes, Actinobacteria, Cyanobacteria*, and *Fusobacteria*, with these phyla representing 99% of the total abundance, and *Firmicutes* alone comprising 97% of total bacteria present. Cecum samples were primarily composed by *Firmicutes, Bacteroidetes, Proteobacteria, Fusobacteria, Cyanobacteria*, and *Verrucomicrobia*, with these phyla representing 96% of all bacteria present. Cloacal samples were dominated by *Firmicutes, Fusobacteria, Proteobacteria, Bacteroidetes, Actinobacteria*, and *Synergistetes*, with these six phyla representing 98% of the total relative abundance. Skin samples were primarily composed by *Firmicutes, Bacteroidetes, Proteobacteria, Actinobacteria, Fusobacteria*, and *Cyanobacteria*; these phyla comprised 98% of the total bacterial community present.

#### Phyla relative abundance comparisons among flocks

*Firmicutes* and *Bacteroidetes* were the two most abundant phyla in cecum across flocks. *Firmicutes* was found at 52.62, 55.27, 50.12% in FUDS+, FUDS-, and control flocks, respectively while *Bacteroidetes* was found at 29.59, 27.52, and 32.97 for FUDS+, FUDS-, and control flocks, respectively.

Ileum samples were dominated by *Firmicutes* with similar relative abundances across flocks and disease state. It was found at 97.10, 94.56, and 96.67% for FUDS+, FUDS- and control flocks, respectively.

Cloacal samples had notable abundance differences across samples. Main differences were observed for the top four phyla: *Firmicutes, Fusobacteria, Proteobacteria*, and *Bacteroidetes*. FUDS+ birds had the highest relative abundance of *Firmicutes* representing 73.18, 62.92, and 66.52% for FUDS +, FUDS-, and control birds, respectively. *Fusobacteria* was found at 13.73, 24.23, and 8.52% across FUDS+, FUDS- and control birds, respectively. *Proteobacteria* displayed a mild trend with FUDS+ birds having the lowest relative abundance when compared to asymptomatic and healthy birds. *Proteobacteria* were present at 4.21, 6.12, and 7.86% in FUDS+, FUDS- and control birds, respectively.

Skin samples microbiota was dominated by *Firmicutes, Bacteroidetes*, and *Proteobacteria*. Across disease state, the skin microbiome from sick birds had higher relative abundances of *Firmicutes* and *Proteobacteria*, and lower relative abundances of *Bacteroidetes* when compared with healthy birds. *Firmicutes* relative abundances were at 80.10, 71.11, and 64.79% for FUDS+, FUDS- and control birds, respectively. *Bacteroidetes* was found at 4.11, 14.84, and 21.31% for FUDS+, FUDS- and control birds, respectively. The relative abundance of *Proteobacteria* was found at 9.87, 4.08, and 3.33% for FUDS+, FUDS- and control birds, respectively.

#### Relative abundance by genera

**Figure 4:**
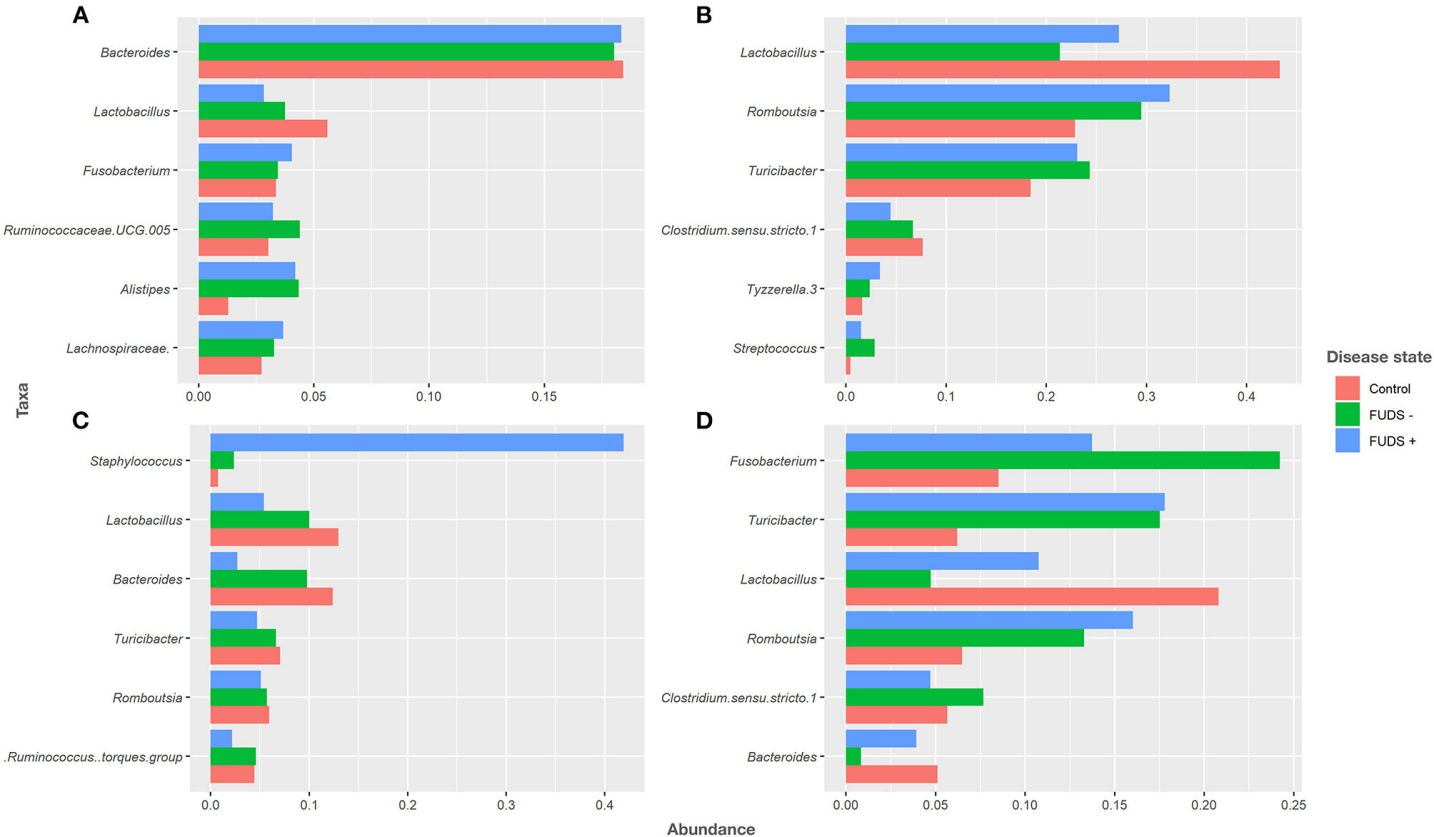
Relative abundance plot of top six taxa at genus level for Control, FUDS –, and FUDS + flocks by sample type. **(A)** Cecum; **(B)** ileum; **(C)** skin; and **(D)** cloaca.

At the genus level for all flocks, the six most predominant genera by sample type are shown in Figure 4. Ileum samples were primarily composed by *Lactobacillus, Rombustia, Turnibacter, Clostridium, Tyzzerella*, and *Streptococcus*, with these genera representing 91% of total bacterial population. Cecum samples were dominated by *Bacteroides, Lactobacillus, Fusobacterium, Ruminococcaceae UCG.005, Alistipes*, and *Lachnospiraceae*, with these genera representing 36% of total bacteria. The top six genera from cloacal samples were *Fusobacterium, Turnibacter, Lactobacillus, Rombustia, Clostridium*, and *Bacteroides*, with these genera representing 63% of the total bacteria present. Skin samples were mainly composed by *Staphylococcus, Lactobacillus, Bacteroides, Turnibacter, Rombustia*, and *Ruminococcus*, with these genera representing 48% of the total skin microbiota.

#### Skin samples taxa relative abundance

**Figure 5:**
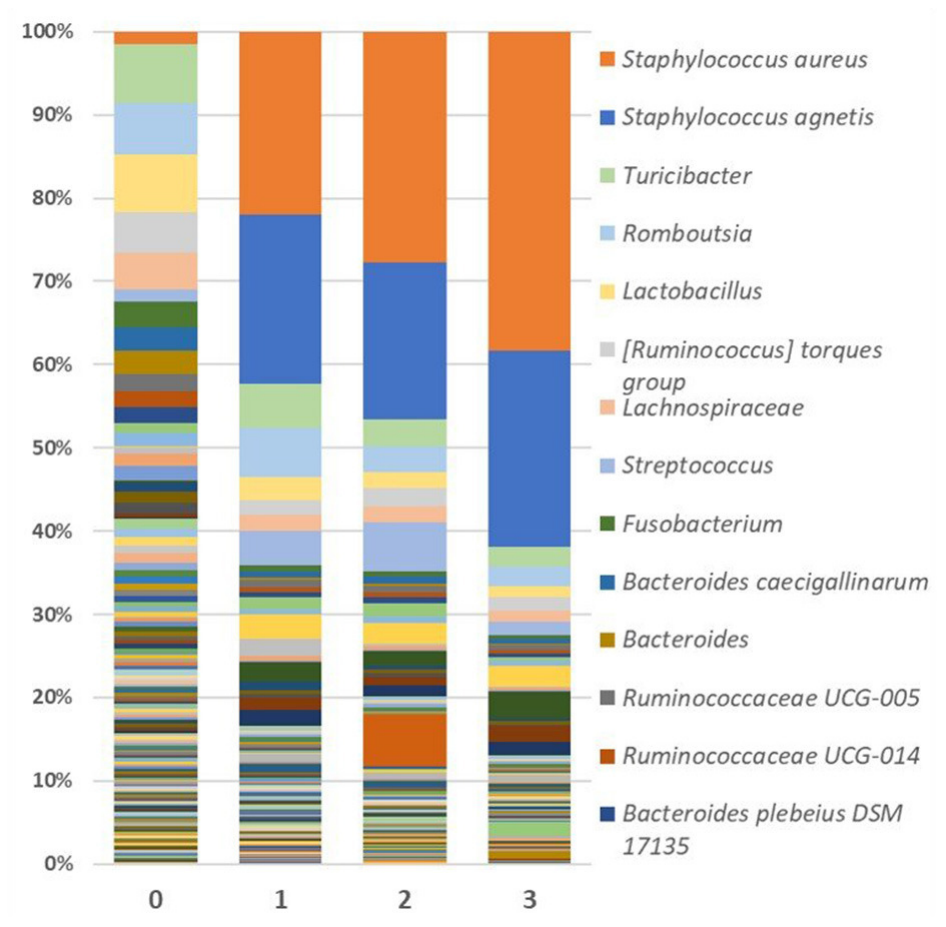
Relative abundance as average percentage of bacterial taxa populations for skin samples grouped by lesion score. The predominant S*taphylococcus aureus* OTU was present at 0.709, 2.063, 21.647, and 37.787% for lesion scores 0, 1, 2, and 3, respectively. *Staphylococcus agnetis* was present at 0.043, 19.969, 18.564, and 23.125% for lesion scores 0, 1, 2, and 3, respectively.

Looking specifically at the lesion, *Staphylococcus* abundance was significantly greater (*p* < 0.001) in the skin of FUDS + birds compared to FUDS–birds, highlighting a potential association with the development of FUDS. The relative abundance by genus from skin samples for control and FUDS flocks is shown in Figure 5. *Staphylococcus spp*. abundance increased as FUDS lesion progressed. In samples from the control flock (lesion score = 0), *Staphylococcus spp*. represented 0.7% of total relative abundance whereas, in the samples from the FUDS+ flock, *Staphylococcus spp*. concentration was 2.1, 41.6, 45.6, and 60.8%, at lesion scores 0, 1, 2, and 3, respectively. *Staphylococcus* diverged into two similarly abundant OTU groups when accessed at the species level. However, Silva taxonomic identification was able to speciate *Staphylococcus agnetis* for one OTU group, whereas the second group was not identified below the genus level. Further analysis *via* NCBI blast of the sequences associated with the second *Staphylococcus* group identified them as *Staphylococcus aureus*. The predominant S*taphylococcus aureus* OTU was present at 0.71, 2.06, 21.65, and 37.79% for lesion scores 0, 1, 2, and 3, respectively. *Staphylococcus agnetis* was present at 0.04, 19.97, 18.56, and 23.13% for lesion scores 0, 1, 2, and 3 respectively.

#### Genera relative abundance comparisons among flocks

*Bacteroides* and *Lactobacillus* were the two most abundant genus in cecum samples across flocks. *Lactobacillus* relative abundance was found at increased levels in control (5.62%), followed by FUDS- (3.77%), and FUDS + (2.82%) birds, respectively. *Bacteroides* relative abundances were similar among flocks, found at 18.35, 18.03, and 18.04% in FUDS+, FUDS- and control birds, respectively.

*Lactobacillus* was the most abundant taxon in the ileum samples with increased abundance in the control samples. *Lactobacillus* was found at 27.24, 21.35, and 43.38% in FUDS+, FUDS-, and control birds, respectively. *Romboutsia* trended downward following disease state, being the highest in FUDS+ birds at 32.36%, followed by 29.474% in FUDS -, and 18.437% in control birds.

Cloacal samples displayed the most differences across flocks and disease state with *Fusobactereium, Turicibacter* and *Lactobacillus* being the top three most abundant taxa. *Fusobacterium* was present at 13.72, 24.23, and 8.46% in FUDS+, FUDS- and control birds, respectively. *Turicibacter* was notably different among flocks, but not across disease state within a flock. *Turicibacter* made up 17.78%, and 17.52% of FUDS+ and FUDS- birds' microbial composition, while only comprising 6.27% of control bird cloacal microbiota. *Lactobacillus* was also differentially abundant across flocks and disease state, with its peak representation in the control flock. *Lactobacillus* was found at 10.75, 4.74, and 21.61% in FUDS+, FUDS- and control birds, respectively.

Skin samples were dominated by *Staphylococcus, Lactobacillus* and *Bacteroides*, with notable trends across disease state and flocks. *Staphylococcus* comprised 41.96% of FUDS+, 2.35% of FUDS-, and 0.77% of control skin samples. *Lactobacillus* exhibited its lowest representation in FUDS+ samples. *Lactobacillu*s was present at 5.39, 10.02, and 13.05% in FUDS+, FUDS-, and control samples, respectively. *Bacteroides* displayed a similar trend as *Lactobacillus* among disease states. *Bacteroides* was found at 2.72, 9.79, 12.01% in FUDS+, FUDS-, and control samples, respectively.

#### Staphylococcus relative abundance by sample type

*Staphylococcus* abundance in the skin was found at 41.96, 2.63, and 0.77%, and for FUDS+, FUDS -, and control flocks, respectively. In the ileum it was at 0.032, 0.052, and 0.011% for FUDS+, FUDS-, and control flocks, respectively. *Staphylococcus* in the cloaca was found at 0.058, 0.075, and 0.654%, for FUDS+, FUDS-, and control flocks, respectively. The lowest *Staphylococcus* abundance was found in the cecum, found at 0.001, 0.003, and 0% for FUDS+, FUDS-, and control flocks, respectively.

#### Linear discriminate analysis effect size analysis

**Figure 6:**
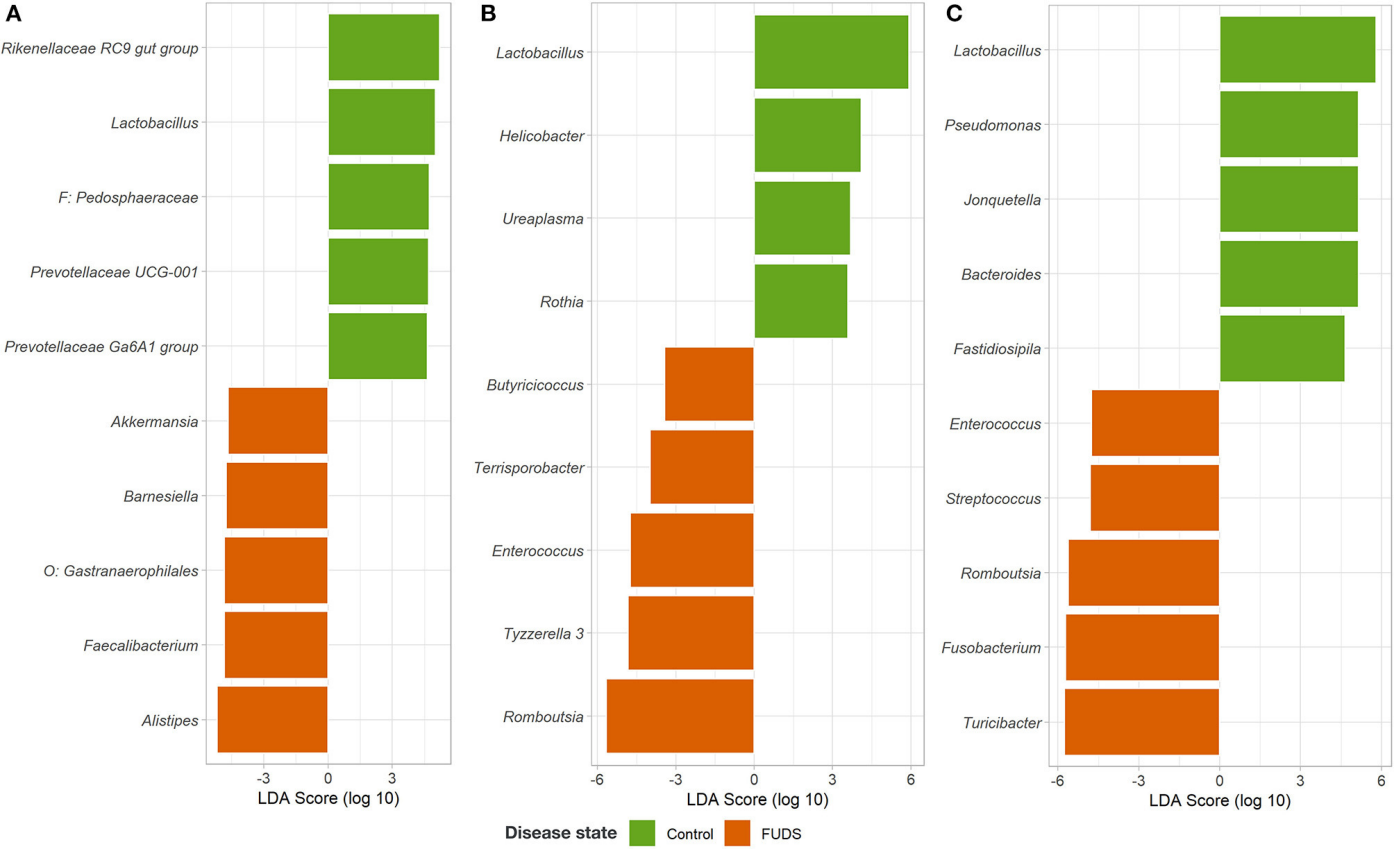
Linear discriminant analysis (LDA) effect size (LEfSe) for microbial differential abundance between Control and FUDS affected flocks. **(A)** Cecum, **(B)** ileum, and **(C)** cloaca.

**Figure 7:**
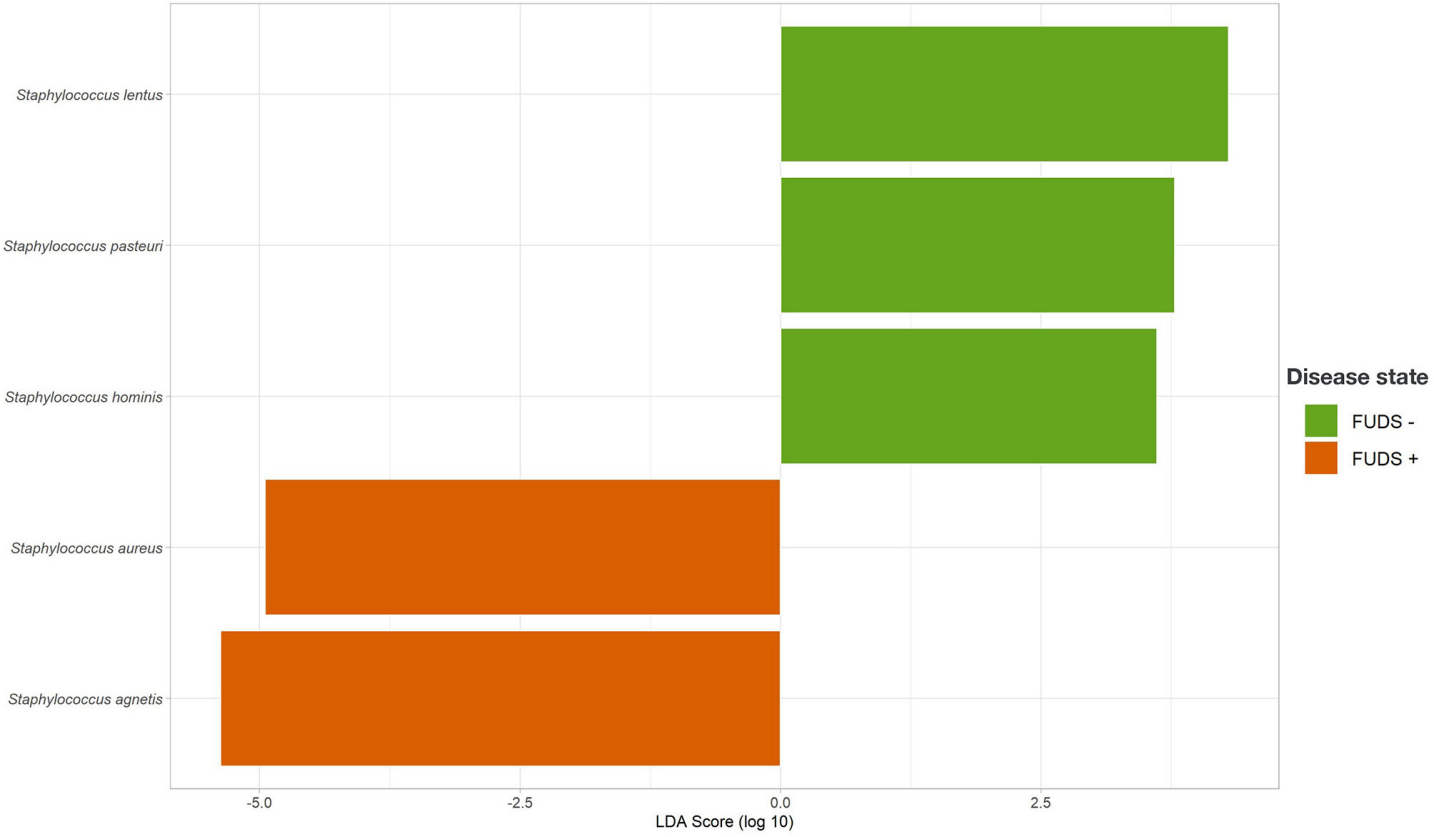
Linear discriminant analysis (LDA) effect size (LEfSe) for *Staphylococcus* differential abundance between FUDS + and FUDS–flocks.

The LEfSe analysis was performed to identify differential microbial features between the Control and FUDS affected flocks (Figure 6). For the skin, the genus *Staphylococcus* was significantly enriched (*p* < 0.05) in FUDS affected flock and *Bacteroides* and *Lactobacillus* significantly enriched (*p* < 0.05) in the Control (healthy) flock. A second LEfSe analysis was performed only on *Staphylococcus* to identify specific species associated with the health status of the birds (Figure 7). *S. aureus* and *S. agnetis* were identified as the only two taxa associated with FUDS + birds (*p* < 0.05), while *S. cohnii* was significantly enriched in the Control flock. *Lactobacillus* was found as a significant taxon associated with the healthy group in all sample types analyzed in this study. Further LEfSe analysis on the differential *Lactobacillus spp*. showed a high diversity of *Lactobacillus* associated with healthy birds, with *L. alvi, L. gallinarum, L. johnsonii*, and *L. acidophilus* differentially abundant in the control flock as compared to the FUDS affected flock.

### Whole genome sequence analysis

**Figure 8:**
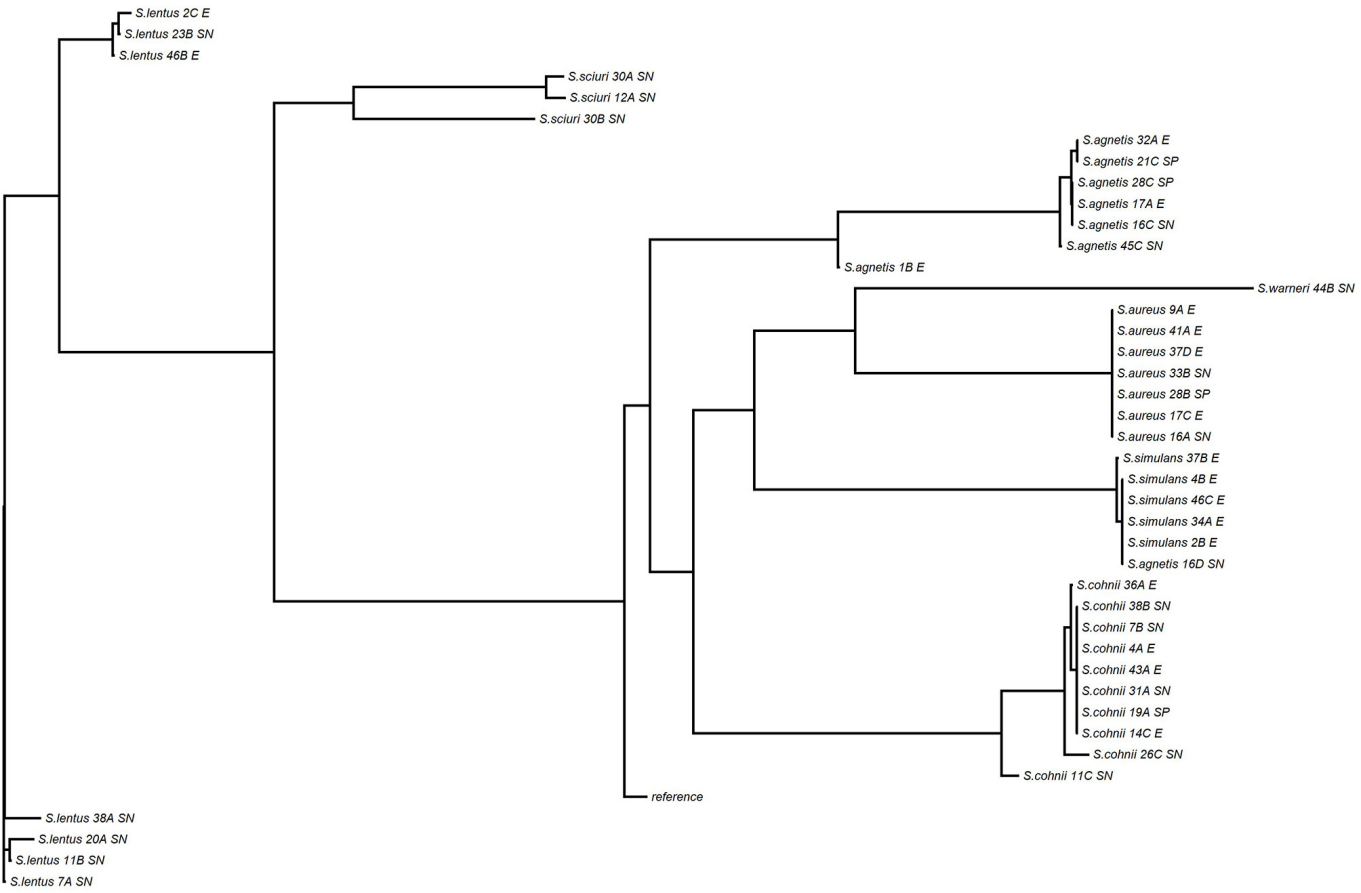
Phylogenetic tree of *Staphylococcus* isolates from skin and environmental samples of Control and FUDS flocks.

The WGS data set analyzed in this study is available in the NCBI Sequence Read Archive repository with accession BioProject ID PRJNA884648. *Staphylococcus* species of all 68 isolates selected were confirmed by WGS, three isolates taxonomically identified by 16S PCR amplification as *S. fleuretti, S. simulans*, and *S. pasteuri* were identified by WGS as *S. sciuri, S. aureus*, and *S. warneri*, respectively (Table 3, Figure 8).

**Table 3:** Whole genome sequencing analysis of staphylococci isolates from control and FUDS affected flocks, bioinformatics analysis for identificantion of AMR genes.

**Strain ID**	**Species**	**Flock**	**Source**	**Disease state**	**Antimicrobial phenotype**	**Antimicrobial resistance (AMR) genes**							
						**MLS**	**Aminoglycoside**	**Tetracycline**	**Streptogramin A/** **Pleuromutilin/** **Lincosamide**	**Macrolide**	**Aminocyclitol**	**Beta-lactam**	**QACs**
7A	*S. lentus*	Control	Skin	*Negative*	Macrolide resistance	*mph*(C)							
7B	*S. cohnii*	Control	Skin	*Negative*	Macrolide, Lincosamide and Streptogramin B, Amonglycosidase	*msr*(A)*, lnu(A)*	*str*						
11B	*S. lentus*	Control	Skin	*Negative*	Macrolide, Lincosamide, Tetracycline resistance	*msr*(A)*, lnu(A)*		*tet*(K)					
11C	*S. cohnii*	Control	Skin	*Negative*	Tetracycline resistance			*tet*(K)					
12A	*S. sciuri*	Control	Skin	*Negative*	Streptogramin A, Pleuromutilin, and Lincosamide resistance				*sal* (A)			*mec* A1	
45C	*S. agnetis*	Control	Skin	*Negative*	No antimicrobial resistance								
5A	*S. cohnii*	Control	Skin	*Negative*	Quaternary Ammonium Compounds resistance, macrolide, lincosamide and streptogramin B resistance	*msr*(A)				*mph*(C)			*qac*G
6A	*S. cohnii*	Control	Skin	*Negative*	No antimicrobial resistance								
8A	*S. cohnii*	Control	Skin	*Negative*	Quaternary Ammonium Compounds resistance, macrolide, lincosamide and streptogramin B resistance	*msr*(A)				*mph*(C)			*qac*G
10C	*S. cohnii*	Control	Skin	*Negative*	Macrolide, Lincosamide and Streptogramin B resistance, tetracycline, quaternary ammonium compounds resistance	*msr*(A)		*tet*(K)					*qac*G
12D	*S. cohnii*	Control	Skin	*Negative*	Aminoglycoside resistanc, macrolide resistance, quaternary ammonium compounds resistance		*str*			*mph*(C)			*qac*G
15B	*S. cohnii*	Control	Skin	*Negative*	Aminoglycoside resistance, quaternary ammonium compounds resistance		*str*						*qac*G
1B	*S. agnetis*	Control	Environmental	*NA*	Lincosamide, quaternary ammonium compound resistance	*lnu*(A)							*qac*G
2B	*S. simulans*	Control	Environmental	*NA*	Aminoglycoside resistance		*str*						
2C	*S. lentus*	Control	Environmental	*NA*	No antimicrobial resistance								
4A	*S. cohnii*	Control	Environmental	*NA*	Macrolide, Lincosamide and Streptogramin B resistance	*msr*(A)							
4B	*S. simulans*	Control	Environmental	*NA*	Tetracycline, QACs, Aminoglycoside		*str*	*tet*(K)					*qac*G
9A	*S. aureus*	Control	Environmental	*NA*	Quaternary Ammonium Compounds resistance								*qac*G
34A	*S. simulans*	Control	Environmental	*NA*	No antimicrobial resistance								
43A	*S. cohnii*	Control	Environmental	*NA*	Macrolide and Streptogramin B resistance	*msr*(A)		*tet*(K)					*qac*G
2D	*S. agnetis*	Control	Environmental	*NA*	No antimicrobial resistance								
9B	*S. lentus*	Control	Environmental	*NA*	No antimicrobial resistance								
16A	*S. aureus*	FUDS	Skin	*Negative*	Quaternary Ammonium Compounds resistance								*qac*G
16C	*S. agnetis*	FUDS	Skin	*Negative*	No antimicrobial resistance								
16D	*S. simulans*	FUDS	Skin	*Negative*	No antimicrobial resistance								
23B	*S. lentus*	FUDS	Skin	*Negative*	No antimicrobial resistance								
26C	*S. cohnii*	FUDS	Skin	*Negative*	Macrolide and Streptogramin B resistance	*msr*(A)				*mph*(C)			*qac*G
30A	*S. sciuri*	FUDS	Skin	*Negative*	Streptogramin A, Pleuromutilin, and Lincosamide resistance				*sal* (A)			*mec* A1	
30B	*S. sciuri*	FUDS	Skin	*Negative*	Quaternary Ammonium Compounds resistance							*mec* A	*qac*G
31A	*S. cohnii*	FUDS	Skin	*Negative*	Macrolide and Streptogramin B resistance	*msr*(A)		*tet*(K)		*mph*(C)			
20A	*S. lentus*	FUDS	Skin	*Negative*	Macrolide resistance								
33B	*S. aureus*	FUDS	Skin	*Negative*	No antimicrobial resistance								
38A	*S. lentus*	FUDS	Skin	*Negative*	Macrolide resistance					*mph*(C)			
38B	*S. cohnii*	FUDS	Skin	*Negative*	Macrolide and Streptogramin B resistance	*msr*(A)							*qac*G
44B	*S. warneri*	FUDS	Skin	*Negative*	Tetracycline resistance		*str*	*tet*(K)					
26B	*S. lentus*	FUDS	Skin	*Negative*	Macrolide resistance					*mph*(C)			
33D	*S. cohnii*	FUDS	Skin	*Negative*	Macrolide, Lincosamide and Streptogramin B resistance, quaternary ammonium compounds resistance	*msr*(A)							*qac*G
19A	*S. cohnii*	FUDS	Skin	*Positive*	Macrolide and Streptogramin B resistance	*msr*(A)	*str*						
21C	*S. agnetis*	FUDS	Skin	*Positive*	No antimicrobial resistance								
28B	*S. aureus*	FUDS	Skin	*Positive*	No antimicrobial resistance								
28C	*S. agnetis*	FUDS	Skin	*Positive*	No antimicrobial resistance								
18A	*S. agnetis*	FUDS	Skin	*Positive*	Quaternary Ammonium Compounds resistance								*qac*G
18B	*S. agnetis*	FUDS	Skin	*Positive*	Quaternary Ammonium Compounds resistance								*qac*G
21B	*S. aureus*	FUDS	Skin	*Positive*	Quaternary Ammonium Compounds resistance								*qac*G
22B	*S. aureus*	FUDS	Skin	*Positive*	Quaternary Ammonium Compounds resistance								*qac*G
24B	*S. aureus*	FUDS	Skin	*Positive*	Quaternary Ammonium Compounds resistance								*qac*G
24D	*S. agnetis*	FUDS	Skin	*Positive*	No antimicrobial resistance								
25A	*S. agnetis*	FUDS	Skin	*Positive*	No antimicrobial resistance								
25B	*S. agnetis*	FUDS	Skin	*Positive*	No antimicrobial resistance								
27A	*S. aureus*	FUDS	Skin	*Positive*	No antimicrobial resistance								
27B	*S. agnetis*	FUDS	Skin	*Positive*	No antimicrobial resistance								
29A	*S. aureus*	FUDS	Skin	*Positive*	No antimicrobial resistance								
39B	*S. aureus*	FUDS	Skin	*Positive*	No antimicrobial resistance								
14C	*S. cohnii*	FUDS	Environmental	*NA*	Macrolide and Streptogramin B resistance	*msr*(A)							
17A	*S. agnetis*	FUDS	Environmental	*NA*	No antimicrobial resistance								
17C	*S. aureus*	FUDS	Environmental	*NA*	Quaternary Ammonium Compounds resistance								*qac*G
32A	*S. agnetis*	FUDS	Environmental	*NA*	No antimicrobial resistance								
36A	*S. cohnii*	FUDS	Environmental	*NA*	Macrolide, Lincosamide and Streptogramin B resistance	*erm*(A)		*tet*(K)			*ant* (9)-Ia	*bla*Z	
37B	*S. simulans*	FUDS	Environmental	*NA*	Beta-lactam resistance							*bla*Z	
37D	*S. aureus*	FUDS	Environmental	*NA*	No antimicrobial resistance								
41A	*S. aureus*	FUDS	Environmental	*NA*	No antimicrobial resistance								
46B	*S. lentus*	FUDS	Environmental	*NA*	No antimicrobial resistance								
46C	*S. simulans*	FUDS	Environmental	*NA*	No antimicrobial resistance								
14B	*S. agnetis*	FUDS	Environmental	*NA*	No antimicrobial resistance								
32D	*S. agnetis*	FUDS	Environmental	*NA*	No antimicrobial resistance								
37A	*S. cohnii*	FUDS	Environmental	*NA*	Macrolide, Lincosamide and Streptogramin B resistance	*msr*(A)							
37C	*S. lentus*	FUDS	Environmental	*NA*	No antimicrobial resistance								
42A	*S. aureus*	FUDS	Environmental	*NA*	No antimicrobial resistance								

#### Characterization of antimicrobial resistance and disinfectant resistance

A total of 30 *Staphylococcus* isolates (44.12%) out of the 68 sequenced had between one and four acquired AMR genes encoding for macrolides, lincosamides, spectrogramines, aminoglycosides, tetracyclines, and beta-lactams resistance (Table 3). The *msr*A gene was the most common AMR acquired gene identified in the set, encoding for macrolide, lincosamide and streptogramin B resistance. It was carried by 14 isolates (13 *S. cohnii* isolates, 1 *S. lentus*) and was found alone (*n* = 6) or in combination with *mph*C (*n* = 3)*, tet*K (*n* = 2)*, lnu*A (*n* = 2), and *spr* (*n* = 1). The *tet*K and *mph*C genes encoding the tetracycline efflux protein and the macrolide phosphotransferase C protein, respectively, were the second and third most common acquired AMR genes identified carried by 9 (5 *S. cohnii*, 2 *S. lentus*, 1 *S. warneri*, and 1 *S. simulans*) and 8 (5 *S. cohnii*, 3 *S. lentus*) isolates, respectively. The *tet*K gene was found alone (*n* = 1), or in combination with *msr*A (*n* = 4), *str* (*n* = 2), and *mph*C (*n* = 2). The *mph*C gene was found alone (*n* = 2), or in combination with *msr*A (*n* = 4), *tet*K (*n* = 1), and *str* (*n* = 1).

##### Control flock

A total of 22 *Staphylococcus* isolates (32.35%) out of the 68 sequenced were isolated from skin (*n* = 12; 54.45%) and from environment (*n* = 10; 45.45%). Ten (83.33 %) of the skin isolates had AMR genes in their genomes, with the *mph*C, *msr*A, and *lnu*A genes associated with MLS (macrolide, lincosamide and streptogramin B) resistance (*n* = 6), as the most prevalent; 2 isolates (16.67%) did not have AMR genes in their genomes. Five (50%) of the environmental isolates had AMR genes associated with MLS, aminoglycoside, and tetracycline resistance, the 5 remaining environmental isolates had no AMR genes in their genomes (Table 3).

##### FUDS affected flock

A total of 46 *Staphylococcus* isolates (67.64%) out of the 68 sequenced were isolated from FUDS–skin (*n* = 15; 32.61%), FUDS + skin (*n* = 16; 34.78%), and from environment (*n* = 15; 32.61%). Differences in AMR genes content was observed among FUDS–and FUDS + skin isolates. A total of 11 (73.33%) FUDS–skin isolates had AMR resistance genes associated with MLS and tetracycline resistance, with the *msr*A as the most common; the 4 (26.77%) remaining isolates had no AMR resistance genes in their genomes. FUDS + skin isolates were dominated by *S. aureus* and *S. agnetis*, all *S. aureus* (*n* = 7) and *S. agnetis* (*n* = 8) selected for WGS had no AMR genes in their genomes. One isolate, *S. cohnii*, from FUDS+ skin samples had the *msr*A gene. The environmental isolates selected were composed of *S. aureus* (*n* = 4), *S. agnetis* (*n* = 4), *S. cohnii* (*n* = 3), *S. simulans* (*n* = 2), *S. lentus* (2). A total of 11 (73.33%) of the selected isolates had no AMR genes in their genomes, similarly to the skin + isolates, *S. aureus* and *S. agnetis* isolates did not have AMR genes in their genomes. The 4 (26.67%) remaining environmental isolates had AMR genes associated with MLS, tetracycline, and beta-lactam resistance (Table 3).

#### Disinfectant resistance

A total of 20 isolates (29.41%), out of the 68 sequenced, had the *qac*G gene encoding resistance to quaternary ammonium compounds (QACs). The QACs resistant isolates were composed of 5 (25%) from skin in the control group, 4 (20%) from environment of control group, 5 (25%) from FUDS + skin, 5 (25%) from FUDS–skin, and 1 (5%) from FUDS house environment. Eight (40%) isolates had the *qua*G gene alone, the 12 (60%) remaining had the *qua*G gene in combination with one (*n* = 5), or two (*n* = 7) acquired AMR genes. The most common phenotype identified in the isolates exhibiting QACs resistance was *msr*A- *mph*C- *qac*G (*n* = 3), *msr*A- *tet*K- *qac*G (*n* = 2), *msr*A- *qac*G (*n* = 2) (Table 3).

#### Identification of Staphylococcus virulence factors

Six classes of *Staphylococcus* VF were identified including adherence, enzyme, immune evasion, secretion system, toxin, and iron uptake. In addition, VF with >50% homology to non-*Staphylococcus* species were identified; the latter included the following 7 VF classes: adhesion proteins, regulation, surface protein anchoring, anti-phagocytosis, intracellular survival, serum resistance, and phagosome arresting (Supplementary Table 1).

The VF class with most genes identified were enzyme (*n* = 171) and immune evasion, followed by adherence (*n* = 84), and toxin (*n* = 84). Among the enzymes-encoding genes, *geh* and *lip*, encoding for lipase, were the most prevalent (*n* = 54), followed by *ssp*A (*n* = 39), encoding for serine V8 protease, and *hys*A (*n* = 26), encoding for hyaluronate lyase (Supplementary Table 1). The *geh* and *lip* genes were identified in all *Staphylococcus* species analyzed in this study except for *S. sciuri* and *S. lentus, ssp*A was found across all *Staphylococcus* species, and *hys*A only in *S. aureus* and *S. agnetis* isolates. In the immune evasion category, the most common VF included *cap*B (*n* = 36), encoding for polyglutamic acid capsule, *ads*A (*n* = 35) encoding for adenosine synthase (A), and *gal*E (*n* = 22) encoding for polysaccharide capsule. The *cap*B was found in all *Staphylococcus* species analyzed in this study except for *S. aureus, S. sciuri* and *S. lentus*; *ads*A was only present in *S. aureus, S. agnetis*, and *S. simulans*, and *gal*E present in all species except for *S. aureus, S. sciuri* and *S. warneri*.

In the category of adherence, the *ebp* gene encoding for elastin binding protein was the most common (*n* = 19), followed by *ica*A, *ica*B, and *icaC*, genes associated with intercellular adhesion (*n* = 17), other genes found were *fnb*B (*n* = 13) encoding for fibronectin binding protein, *cna* (*n* = 13) associated with collagen adhesion, and *atl* (*n* = 10) encoding for autolysin. The *ebp* was found only in *S. aureus*, and *S. cohnii*; the *ica*A, *ica*B, and *icaC* genes were only present in *S. aureus, S. sciuri*; the *cna* genes was present only in *S. agnetis*, and the *atl* only present in *S. aureus*. In the category of toxin, the *set* genes encoding for exotoxin were the most common (*n* = 25), followed by *hlg*A*, hlg*B, and *hlg*C genes associated with gamma hemolysin (*n* = 14), and *hlb* (*n* = 14) encoding for hemolysin B. The *set* genes were found only in *S. aureus* and *S. agnetis*. The *hlg*A*, hlg*B, and *hlg*C genes were found only in *S. aureus*; *ads*A was only present in *S. aureus, S. agnetis*, and *S. simulans*, and *gal*E present in all species except for *S. aureus, S. sciuri* and *S. warneri*.

### Antimicrobial effect by agar-well diffusion assay

The inhibitory results of the four proprietary *Bacillus* DFM combinations against *S. aureus* and *S. agnetis* isolated from skin lesions of birds affected with FUDS are shown in Table 4. All DFM tested in this study were found inhibitory against both *Staphylococcus aureus* and *Staphylococcus agnetis. S. aureus* inhibition ranged from 6 to 22 mm with DFM Combo 4 and DFM Combo 1, respectively. *S. agnetis* inhibition ranged from 14.5 to 25 mm with DFM Combo 4 and DFM Combo 1, respectively. *Bacillus* DFM Combo 1 produced the highest inhibitory effect with clear zones of inhibition ranging from 20 to 22 mm for *S. aureus* isolates and 22–25 mm for *S. agnetis* isolates (Table 4). DFM Combo 4 was the least inhibitory with zones of inhibitions ranging from 6 to 11.5 mm for *S. aureus* isolates and 14.5 to 16 mm for *S. agnetis* isolates recovered from the lesions.

**Table 4:** Antimicrobial activity, by agar-well diffusion, of proprietary DFM combination against *S. agnetis* and *S. aureus* isolated from skin of FUDS + birds.

**DFM combos**	**Zone of inhibition averaged from duplicate wells (mm)** ^*^								
	***S. aureus*** **21A**	***S. aureus*** **21B**	***S. agnetis*** **21C**	***S. aureus*** **21D**	***S. aureus*** **24A**	***S. aureus*** **24B**	***S. agnetis*** **24C**	***S. aureus*** **24D**	**Overall score** ^a^
Combo 1	22	20	25	22	20	21	22	20	172
Combo 2	20.5	19	14	20	18	16.5	16	16.5	140.5
Combo 3	15	16.5	17	16.5	15	14	16.5	15	125.5
Combo 4	9	11.5	14.5	10.5	6	10.5	16	11	89

* Low inhibition: 6–10 mm; medium inhibition: 10–14 mm; high inhibition: >14 mm.

a Overall score summed for inhibitions produced by each DFM combo across all Staphylococcus isolates tested.

### Antimicrobial effect by competitive exclusion on broth

The antimicrobial effect of the 4 *Bacillus* DFM combinations by competitive exclusion on broth is shown in Table 5. All DFM combos were found inhibitory, *S. aureus* reductions ranged from 0.24 to 1.55 log_10_ CFU/mL with DFM Combo 4 and Combo 2, respectively. *S. agnetis* and *S. aureus* reductions (log_10_ CFU/mL) were evaluated 24 h after co-inoculation with respect to control samples. Reductions of *S. agnetis* ranged from −0.05 to 1.17 log_10_ CFU/mL with DFM Combo 4 and Combo 2, respectively. Reductions were summed up across all *Staphylococcus* isolates and DFM combos were ranked from highest to lowest antimicrobial effect, DFM combo 2 showed the highest inhibitions across all *Staphylococcus* isolates tested.

**Table 5:** *S. agnetis* and *S. aureus* reduction (log_10_ CFU/ml) by DFM combos co-cultivated 24 h on nutrient broth.

**Log 10 CFU/ml**									
**DFM Combos** ^a^	***S. aureus*** **21A**	***S. aureus*** **21B**	***S. agnetis*** **21C**	***S. aureus*** **21D**	***S. aureus*** **24A**	***S. aureus*** **24B**	***S. agnetis*** **24C**	***S. aureus*** **24D**	**Overall score** ^b^
Combo 2	0.72	0.52	1.05	1.55	0.63	0.95	0.59	1.23	7.24
Combo 3	0.68	0.38	1.17	1.17	0.68	0.84	0.67	0.96	6.53
Combo 1	0.34	0.42	0.63	0.96	0.59	0.73	0.37	0.39	4.43
Combo 4	0.20	0.09	0.55	0.74	0.52	0.56	−0.05	0.53	3.14

a DFM combos ranked from highest to lowest reduction.

b Overall score summed for reductions (log_10_ CFU/ml) produced by each DFM combo across all Staphylococcus isolates tested.

Agar-well diffusion (AWD) overall score was added to the competitive exclusion (CE) overall score to rank the DFM combinations with highest antimicrobial effect (Table 6). DFM Combo 1 had the greatest effect across all isolates tested.

**Table 6:** Combined well-diffusion and competitive exclusion scores of DFM screened across all *Staphylococcus* isolates tested.

**DFM combos**	**Zone of inhibition (mm)**										
	***S. aureus*** **21A**	***S. aureus*** **21B**	***S. agnetis*** **21C**	***S. aureus*** **21D**	***S. aureus*** **24A**	***S. aureus*** **24B**	***S. agnetis*** **24C**	***S. aureus*** **24D**	**Overall AWD Score** ^a^	**Overall CE Score** ^b^	**Overall Score** ^c^
Combo 1	22	20	25	22	20	21	22	20	172	4.43	176.43
Combo 2	20.5	19	14	20	18	16.5	16	16.5	140.5	7.24	147.74
Combo 3	15	16.5	17	16.5	15	14	16.5	15	125.5	6.53	132.03
Combo 4	9	11.5	14.5	10.5	6	10.5	16	11	89	3.14	91.14

a AWD, Agar-well diffusion assay.

b CE, Competitive exclusion broth assay.

c Overall score summed for AWD and CE results across all Staphylococcus isolates tested.

## Discussion

The transition from conventional to cage-free production systems has presented some challenges for the egg producers, one of these challenges is the increase of bacterial infections which have been described as one of the most common causes of mortality in birds raised in cage free and litter-based systems (5). Skin infections in commercial poultry have severe impacts on growth, egg production, feed conversion, and overall performance. Members of the *Staphylococcus* genus have been described as common causative agents of these conditions (30, 31). Sources of infectious agents include soil, litter, cross-contamination among infected birds, and fomites such as contaminated equipment.

In this study, next generation sequencing was used to characterize the microbiome composition of sites in the gastrointestinal tract (GIT) and skin of laying hens with signs of focal ulcerative dermatitis (FUDS) and compare it to the microbiome composition of healthy birds with the overall objective of identifying the causative agents of FUDS and potential routes of infection. The comparison of the microbial community structure between healthy and sick populations is crucial to identify what constitutes a healthy gut/skin microbiota and identify potential dysbiosis, thereby providing opportunities to modulate it and improve overall animal health (32). The microbial composition can be affected by many factors including age, breed, diet, sample type (i.e., GIT site), disease state, housing system, and management. A significant variation in the microbial population was observed in this study attributed to health status of the flock and sample type. For example, a genus identified at significantly higher abundance in the skin and confirmed as present in the FUDS + birds was *Staphylococcus. Staphylococcus* abundance in the skin was found at 2.63 and 0.77% in FUDS–and control flocks, respectively, in comparison with FUDS + flock (>41.96%).

Staphylococci are ubiquitous in poultry farms and hatcheries and are natural inhabitants of the chicken skin and mucous membranes but have also been associated with secondary infections (12). The 16S metagenomics and isolation results in this study showed a high diversity of *Staphylococcus* species on skin and environmental samples from FUDS negative and control flocks as compared to the FUDS positive group. In agreement with the ubiquitous nature of Staphylococci in the microbial population of poultry houses, the environmental samples analyzed in this study showed high diversity of *Staphylococcus* species including *S. cohnii, S. lentus, S. simulans, S. agnetis*, and *S. aureus*. Recent studies have associated the abundance of coagulase-negative staphylococci (CoNS) such as *S. conhii* and *S. simulans* with healthy skin in humans and mice models (33–35). In 2020, Brown et al. (34) reported that the commensal *S. simulans* effectively blocked methicillin-resistant *Staphylococcus aureus* (MRSA) quorum sensing in an infection model in mice. The observed resistance to pathogenic colonization was a result of the production of an autoinducing peptide that acted as MRSA *S. aureus* accessory gene regulator (*agr*) inhibitor protecting the host from invasive infection. In this study, *S. simulans* was isolated from all environmental sample types and was also isolated from skin samples of FUDS negative and control groups. This result could suggest a potential protective effect of this species in the development of the disease.

*Staphylococcus cohnii* was another commensal Staphylococci identified on samples from the skin of FUDS negative and control birds and not present on FUDS positive skin samples. It was also identified by the LEfSe analysis as the most enriched taxon in the Control flock (healthy) not present in the FUDS flock. Similar to *S. simulans, S. cohnii* has been associated with healthy skin. In a skin microbiome analysis of mice with dermatitis found that commensal *S. cohnii* isolated from sick mice had preventive and therapeutic effects on *S. aureus* mediated skin inflammation (33). Further investigation is needed to determine the potential protective effect of commensal Staphylococci found on control and FUDS- samples and not isolated from FUDS + skin samples, on the development of FUDS.

The *Lactobacillus* genus was found ubiquitously in all sample types associated with the healthy birds. Our findings are similar to those observed by Ngunjiri et al. (36) who studied the effect of age and body site in the microbial composition of commercial layer chickens. The authors found *Lactobacillus* ubiquitous in the trachea, nasal, ileum, and cecum samples analyzed. *Lactobacillus* was the most significant taxon in the cloaca and ileum of the control flock, suggesting a potential beneficial/protective effect for the birds. *Lactobacillus* has been commonly ascribed a protective effect through the production of lactic acid that promotes the inhibition of pathogenic bacteria.

The contribution of the skin microbiota to the development of FUDS was analyzed through 16S metagenomic sequencing, and the results showed a higher *Staphylococcus* load (>60% of all bacteria present) as skin lesions progressed from lesion score 0 to 3 than on FUDS negative and control samples. The dysbiosis revealed was characterized by increased abundance of *S. aureus* and *S. agnetis*. These results were confirmed by plate count enumeration with *S. aureus* and *S. agnetis* being the only Staphylococci isolated from the skin of FUDS positive birds. Our results agree with those observed by Ito et al. (33) in their comparative skin microbiome analysis in mice to identify commensal strains with protective effect of inflammatory skin diseases. The authors observed that the severity of dermatitis in mice was associated with an increased abundance of *S. aureus* and *Corynebacterium mastitidis* and negatively associated with the abundance of *S. cohnii* (33).

*Staphylococcus aureus* has been associated with the development of multiple localized infections in poultry including bumblefoot, joint infections, osteomyelitis, and skin abscesses (9, 37, 38). It is also the most common non-clostridial bacterial pathogen associated with gangrenous dermatitis, a systemic infection in chickens (31). In our study, *S. aureus* isolates were recovered from all environmental samples and skin samples from FUDS + birds which demonstrates broad distribution of the pathogen in the poultry house tested. Meyer et al. (9) reported that *S. aureus* was the main bacterial pathogen isolated from different tissues (lungs, liver, wattle lesions, among others) from multiple commercial layer hen flocks, experiencing an increased mortality and a drop in egg production between April and November of 2018. Even though, *S. aureus* was the predominant bacteria isolated, a challenge study, to elucidate the mechanisms by which *S. aureus* could have been causing the systemic infection, was not able to replicate the disease, highlighting the potential role of other infectious agents in the development of the disease (9). In our study, *S. aureus* and *S. agnetis* were found in combination in skin samples from FUDS + birds, suggesting a potential synergistic interaction of both staphylococci in the pathogenesis of the disease.

*Staphylococcus agnetis*, a coagulase-variable *Staphylococcus*, was identified as a separate species in 2012, associated with clinical and subclinical cases of bovine mastitis (39). Since its discovery, it has been recognized as the causative agent of poultry diseases such as bacterial chondronecrosis with osteomyelitis (BCO) (40), valvular endocarditis and septicemia in broiler breeder flocks (41). In our study, *S. agnetis* isolates were recovered from environmental samples from control (nest box, scratch) and FUDS (nest box) houses; similar to *S. aureus*, it was positively correlated with the severity of the lesion with increased abundance in samples from FUDS + birds with a lesion score 3. *S. agnetis* isolates were found clustered together, suggesting a single clone circulating in the farm environment.

FUDS is an emerging disease of cage-free laying flocks, cases have been identified in both Bovans and Hy-Line browns. Previous bacterial analyses on swabs from FUDS lesions identified *S. hyicus*, a skin commensal bacterium associated with opportunistic infections, as the main pathogen present in lesions (42). In our study, 16S microbiome analysis and bacterial isolation of isolates from the skin, identified *S. agnetis* and *S. aureus* in FUDS positive birds. The WGS of *S. agnetis* isolates recovered confirmed them as *S. agnetis*. As identified by Taponen and others (39), *S. agnetis* isolates are closely related to *S. hyicus* and *S. chromogenes*, their differentiation using phenotypic methods and 16S PCR amplification has not been successful leading to misidentification of these species (8). In addition, it is hypothesized that *S. agnetis* diverged from *S. hyicus* and that some of the infections attributed to *S. hyicus* might have been caused by *S. agnetis* (8).

Further characterization to identify antimicrobial resistance genes and virulent factors that could have contributed to the development of FUDS was performed on a selected set of *Staphylococcus* isolates recovered in this study. The use of antibiotics in livestock for disease control and growth promotion has led to the emergence of AMR genes and a worldwide public health concern, due to their potential dissemination to commensal bacteria (43). Antimicrobial resistance genes were widely spread in the screened isolates, with 44.12% of them harboring between one and four acquired AMR genes, they were found equally distributed in control and FUDS flocks, in the skin and environmental samples. Macrolide, lincosamide and spectrogramine resistance was the most common, characterized by the presence of the *msrA* and *mphC* genes in the isolates' genotypes, the *msrA* gene was harbored by 46.66% of the isolates (13 *S. cohnii* isolates, 1 *S. lentus*). Our results are in agreement with those observed by Zmantar and others (44) in a study to determine the antimicrobial susceptibility and resistance to quaternary ammonium compounds (QACs) in clinical *S. aureus* and coagulase negative *Staphylococcus* (CoNS). Authors found the *msrA* gene as one of the most prevalent, identified in 41% of CoNS, and in 10.2% of *S. aureus* strains. Surprisingly, in our study, all *S. aureus* and 93.33% (*n* = 14/15) of *S. agnetis* isolates did not carry any AMR genes in their genotypes. The *msrA* gene encodes for an ATP-dependent efflux pump that transports erythromycin and streptogramin B from the cell inducing resistance to these antibiotics; and has been mostly associated to *Staphylococcus* and recently discovered in other genera, including *Streptococcus, Enterococcus* and *Pseudomonas* (45).

The *tetK* gene was the second most common AMR gene identified in our study, harbored by 30% of the isolates. It encodes a tetracycline efflux pump that confers resistance, this resistance is commonly observed in staphylococci isolates regardless of source due to the widespread of *tetK/tetM* genes (46). Ali Syed et al. (47) found similar results, with the *tetK/tetM* genes present in 58.8% of staphylococci from farmed chickens. Similar to our results, the authors found the genes harbored by CoNS. The wide distribution of AMR genes among staphylococci not associated with the development of FUDS constitutes a potential risk of transmission of AMR genes to pathogenic staphylococci or commensal bacteria, highlighting the importance of monitoring antibiotic resistance in poultry facilities.

The harboring of disinfectant-resistance genes was also analyzed in this study. Disinfectants are widely used in poultry farming and consist of two or more active components with quaternary ammonium compounds (QACs) the most frequently used component (48). The application of subinhibitory concentrations has reduced the bactericidal effect of sanitizers, leading to an increased resistance to QACs-based sanitizers and a potential increase of antibiotic resistance. Resistance to QACs is associated to the presence of the *qac* (*qacA, qacB, qacC, qacD*, and *qacG*) and *smr* genes. In this study, 29.41% of the isolates sequenced had the *qac*G gene present, 40% of the isolates (exclusively *S. aureus* and *S. agnetis*) had the *qac*G gene present and no other AMR gene. The remaining 60% of the Staphylococci isolates screened, had the *qac*G gene present in combination in one or two AMR genes with resistance to macrolides the most common. El Zayed Zaki et al. (49) evaluated the prevalence of *qac* and *smr* genes in clinical isolates of *S. aureus* from hospital-acquired infections and their susceptibility to QACs-based sanitizers and antibiotics. Authors found the *qac*G gene as one of the most prevalent genes, present in 17.8% of the isolates, the results were strongly correlated with reduced susceptibility to QACs-based sanitizers. In our study, *S. aureus* and *S. agnetis* from FUDS + birds did not exhibit resistance to QACs.

The analysis of virulence is an important step when evaluating interventions to inhibit the causative agents of a disease. It is well-established that staphylococci express a wide variety of virulence factors that allow them to evade the immune response and increase their infectivity (50). Staphylococci isolates screened in this study harbored a number of virulence genes encoding for enzyme, immune evasion, adherence, and toxins that could have an additive effect in the development of FUDS. The *geh* and *lip* genes encoding for lipase were the most prevalent enzyme-encoding genes identified, both were found widely spread in *Staphylococcus* isolates screened in this study. Lipases are important factors for colonization and persistence of staphylococci strains in the skin and have a potential role in bacterial growth by the release of host fatty acids during pathogenesis. Delekta et al. (51) demonstrated that the inactivation of the *geh* gene reduced the ability of *S. aureus* strains to utilize host LDLs as a source of exogenous fatty acids, with a potential impact on pathogenesis (51). The *hysA* gene encoding for hyaluronate lyase was another common virulence factor identified, only present in *S. aureus* and *S. agnetis*. Hyaluronidases are bacterial enzymes that cleave the β-1,4 glycosidic bond of hyaluronic acid, a natural substance abundant in the skin, joints, and tissues. These virulence factors are associated with a rapid pathogenic dissemination through tissues causing increased lesion size during infection. Tissues with high concentrations of hyaluronic acid have often been found infected with *S. aureus* (52). Ibberson et al. (52) demonstrated a reduction in tissue damage of the lung of mice when challenged using a *S. aureus hysA* mutant strain, emphasizing the role of *hysA* in *S. aureus* bacterial infection. In our study, the *hysA* gene was widely spread among *S. aureus* and *S. agnetis* isolates from FUDS + birds, suggesting a potential role in their dissemination, pathogenicity, and developing of FUDS.

Staphylococci harbors cell wall proteins involved in adhesion together with tissue and immune defense evasion (50). Cell wall proteins interact directly or indirectly with host receptors to initiate attachment to epithelial cells. This interaction between pathogen and host cells has been found to be species and strain dependent (53). The *S. aureus* protein A encoded by the *spA* gene has been commonly associated with immune response evasion. In our study, it was found in 3 *S. aureus* isolates (2 from FUDS environmental samples and 1 from FUDS + skin sample). Other proteins, such as the *fnbB*, encoding for fibronectin binding proteins and *cna* encoding for collagen adhesin were found only in *S. agnetis* isolates (*n* = 13). Poulsen et al. (41) analyzed the genomes of three *S. agnetis* isolates from birds with valvular endocarditis and identified the *fnbB* gene together with other six virulence factors associated with fibronectin binding proteins. The presence of these genes highlights a potential role in cell adhesion, diffusion, and invasion of host tissues. The production of toxins is another important factor that contributes to pathogenesis by the disruption of eukaryotic membranes, interfering with receptors and causing cell lysis (50). *S. aureus* and *S. agnetis* in this study, harbored genes encoding for exotoxin (*set* genes), alpha and beta-porin forming toxins, and exfoliative toxin type A. These toxins have been implicated in the development of *Staphylococcus-*induced dermatoses, such as acantholytic dermatitis in humans (10), and gangrenous dermatitis in poultry (31). Our results concur with those observed by Al-Rubaye et al. (40) who identified *S. agnetis* as a causative agent of lameness in chickens, and the authors also identified a repertoire of virulence determinants including exfoliative toxin A, fibronectin-binding proteins, and several others associated with adherence, toxin biosynthesis, host immune evasion, and secretion systems. Our findings suggest that the development of FUDS occur as a result of a combination of “inside-out” mechanisms by which both staphylococci translocate across the host tissues using the cooperative virulence factors they express and proliferate in the skin developing typical FUDS lesions. Exposed lesions are a source of cross-contamination to other animals and the environment.

Once pathogens were identified and fully characterized the next step was the development of a customized DFM product to inhibit/reduce their growth. The developed product was based on the use of proprietary novel direct fed microbials. DFM's can be a natural alternative to the subtherapeutic use of antibiotics in animal production. There is an increased interest in their use to reduce the spread of antimicrobial resistance while supporting an improved production efficiency. In this study, four proprietary *Bacillus* combinations were developed based on previous antagonistic screening against multiple pathogens including *Staphylococcus spp*. The antimicrobial effect of the combinations was determined based on the overall reduction of *S. aureus* and *S. agnetis* isolates by agar well diffusion and competitive exclusion antimicrobial screenings. DFM combo 1, a combination of two *Bacillus pumilus*, was found the most effective at inhibiting the growth of both staphylococci isolated from the skin of diseased birds as compared to the other combinations tested. As observed in other studies, there was a strong association between *Bacillus* species and antagonistic effect, with highest inhibitions achieved with the *Bacillus pumilus* combination in DFM combo 1, followed by combo 2 containing two *B. pumilus*, a *B. amyloliquifaciens*, and a *B. subtilis* strain.

Bacilli are endospore-forming bacteria widely used in DFM preparations due to their ability to survive high temperatures, acidic environments, and desiccation, thus increasing their viability during manufacturing and pelleting processes and enhancing their stability in the host' GIT (54, 55). They are known to promote gut health by the production of enzymes, antimicrobial peptides, and other metabolites that inhibit/reduce the growth of bacterial pathogens (56). In our study, the *B. pumilus* combination exerted the improved inhibitions with zones of inhibition ranging between 20–22 mm, and 22–25 mm, for *S. aureus* and *S. agnetis* strains, respectively. These inhibitions were higher than those observed by Ouoba and others (57) who reported that, in an agar spot diffusion assay, *B. pumilus* strains B6 and B10 inhibited indicator *S. aureus* isolates with zones of inhibition < 3 mm, 24 h after incubation (57).

Similar to other Bacilli, *B. pumilus* produce antimicrobial peptides such as bacteriocins and other compounds including amicoumacins with antimicrobial, antifungal, and anti-inflammatory properties (58). Aunpad and Bangchang (59) reported the enhanced antimicrobial effect of the bacteriocin pumilicin 4, against methicillin-resistant *S. aureus* (MRSA). In 2012, Hashimoto and others (60) reported a strong anti-MRSA effect by derivatives of amicoumacins A and B with minimum inhibitory concentrations (MICs) between 0.6 and 256 ug/ml allowing no visible growth of MRSA. In our study, the co-culture of *B. pumilus* combo 1 with *Staphylococcus aureus* and *Staphylococcus agnetis* allowed for reductions between 0.34–0.96 log_10_ CFU/ml, and 0.37–0.63 log_10_ CFU/ml, respectively, after 24 h of co-inoculation. It is important to highlight that the concentrations of the *Bacillus pumilus* tested were found at 10^8^CFU/ml, 24 h after co-inoculation.

The control of pathogens is one of the major interests for the poultry industry from an economic and public health point of view. The *Bacillus* DFM supplementation of layer hen diets has shown improved laying production by enhancing gut health, increasing intestinal fermentation and mineral assimilation, and improving the overall daily feed intake (14, 61, 62). The exact mechanism by which *Bacillus* DFM supplementation inhibits pathogens is not well-understood; the direct inhibition by the production of antimicrobial metabolites or competitive exclusion have been suggested as the main modes of action, other mechanisms such as the enhancement of the intestinal mucosal layer to avoid microbial diffusion across the membrane has also been suggested (14). The targeted *B. pumilus* combination developed for this study, inhibited both pathogens *in vitro* and is being used at different farms with history of FUDS with successful results at: (1) inhibiting the synergistic effect of *S. agnetis* and *S. aureus*, (2) decreasing FUDS mortalities, and (3) improving harvestable eggs (data not shown). Further analysis will be performed to determine the effect of the *Bacillus* DFM supplementation on a potential shift of the microbial population of laying hens at farms previously afflicted with FUDS.

## Conclusion

*Staphylococcus aureus* and *Staphylococcus agnetis* were identified as the causative agents of FUDS in laying hens. Both pathogens were found as statistically significant bacterial species only present in the skin of FUDS + birds, this result was confirmed by plating with both staphylococci as the only pathogens isolated from the skin. Both *S. aureus* and *S. agnetis* had a high number of virulence factors associated with adherence, toxin production, and immune evasion that could have contribute to their pathogenesis and the development of FUDS. The customized *Bacillus pumilus* product developed in this study was found successful at inhibiting both pathogens *in vitro* and is currently being fed at poultry facilities with chickens afflicted with FUDS with improved results at reducing mortality and enhanced egg production.

## Data availability statement

The datasets presented in this study can be found in online repositories. The names of the repository/repositories and accession number(s) can be found in the article/Supplementary material.

## Author contributions

DA, DG, and BT performed all experiments in the study. NE collected samples for analysis, scored birds based on lesion progression, and wrote the sampling collection methodology. DA, ML, and DG contributed to the bioinformatics analyses. TK and CN conceived the study and developed the study design. DA wrote the first draft of the manuscript. DG, BT, KR, and EK contributed to writing. All authors contributed to the editing of the final version of the manuscript.
